# Inhibited Maternal Bone Resorption Suppress Fetal Rat Bone Development During Pregnancy

**DOI:** 10.3389/fcell.2020.00083

**Published:** 2020-02-19

**Authors:** Huanhuan Jia, Li Rao, Kai Kei Miu, Shuangjie Tang, Wei Chen, Guozhu Yang, Yuying Li, Qingnan Li, Jun Chen, Li Lu

**Affiliations:** ^1^School of Life Sciences and Biopharmacy, Guangdong Province Key Laboratory for Biotechnology Drug Candidates, Guangdong Pharmaceutical University, Guangzhou, China; ^2^Guangdong Key Laboratory of Laboratory Animals, Guangdong Laboratory Animals Monitoring Institute, Guangzhou, China; ^3^Development and Regenerative Biology Theme, School of Biomedical Sciences, Faculty of Medicine, The Chinese University of Hong Kong, Hong Kong, China

**Keywords:** alendronate, osteoclast, bone resorption inhibition, pregnancy, fetal bone development

## Abstract

**Objective:**

To determine the relationship between maternal bone resorption and bone development in fetuses.

**Methods:**

Female SD rats were injected with either fluorescent calcium indicator calcein alone or together with tetracycline 1 week before pregnancy, followed by fluorescence detection in fetal tibias 21 days post-treatment. Alendronate was subsequently administered to pregnant rats to inhibit maternal bone resorption, while maternal bone turnover and fetal bone development were both examined.

**Results:**

The maternal fluorescent labeled calcium before pregnancy was found in the fetal tibia. This indicated that the calcium of maternal bones may be released into the maternal circulation through high bone resorption during pregnancy, thereby participating in the fetal bone development. Bone histomorphometry and serum biomarker results showed that Alendronate significantly inhibited maternal bone resorption in pregnant rats when compared to normal pregnant rats. Moreover, the body weight, bone mass, and bone length of the fetuses in the Alendronate group were significantly decreased; while no apparent abnormality in placental morphology was observed. The above results implied that when maternal bone resorption is suppressed, the development of the fetal bone shall also be suppressed.

**Conclusion:**

Calcium in the maternal bone is released into the maternal circulation through bone resorption during pregnancy which represents an important material source in fetal bone development. Therefore, high bone turnover during pregnancy is essential for mammalian embryonic bone development.

## Introduction

Pregnancy is an exhaustive period of persistent calcium demand, especially toward late gestation; the placenta actively transports vast amounts of calcium to allow rapid fetal skeletal mineralization (Hacker et al., 2012). Current research perspectives on maternal calcium and bone metabolism during pregnancy are centered to studying the modes of high calcium absorption, high urinary calcium excretion, and high bone turnover (Ritchie et al., 1998; Black et al., 2000; Prentice, 2000). Maternal bone resorption and bone formation are both increased during pregnancy, which are elevated in clinical studies and animal studies, mainly by measurements of plasma bio-markers (Cross et al., 1995; Naylor et al., 2000, 2003). The increases in bone turnover markers are apparent by early gestation, and their levels rise by 50–200% during pregnancy in human beings. Due to the huge demand for calcium during pregnancy, it has long been assumed that additional calcium supplementation from the diet during pregnancy should be necessary (Shoji et al., 2000; O’Brien et al., 2006). However, heated debates in these theories continued. Accumulating clinical evidence indicates that women having high calcium intake during pregnancy do not increase the development of fetal bones compared to those who have normal calcium intake (Kovacs, 2000; Vargas Zapata et al., 2004). It is implied that no additional calcium intake is required during pregnancy, and the maternal physiological adaptation process is sufficient to maintain the calcium requirement for fetal growth (Prentice, 2000). Nonetheless, those findings did not topple the mainstream concept for the importance in calcium supplementation during pregnancy. The missing link appears to be any direct evidence to demonstrate how liberation of calcium from maternal skeleton shall provide the calcium necessary for fetal bone development, perhaps surprisingly, even in animal studies.

To date, the mechanisms by which calcium is supplied to the fetus have not been fully characterized (Hacker et al., 2012). Indeed, the placenta responds actively to meet fetal needs for mineral by efficiently transporting calcium, phosphorus, and magnesium from maternal circulation (Kovacs, 2014), despite the exact source of this calcium has been argued over the years. In fact, the calcium absorbed from the daily diet and the calcium released by maternal bone mobilization will both enter the maternal circulation. So, presumed that the calcium intake during pregnancy has no positive effect on fetal development, does the calcium released by maternal bone mobilization have any contribution to fetal bone development at all? We aim to tackle this question by conducting experiments in parts. Firstly, we performed a fluorescent labeling of rat maternal bone calcium before pregnancy to investigate if the calcium released from maternal bone calcium stores is directly involved in fetal bone development. Secondly, we inhibited the maternal bone resorption of the pregnant rats by Alendronate, so as to explore whether reducing maternal bone resorption shall affect fetal bone development.

Alendronate is the most commonly prescribed bisphosphonates to limit bone turnover via repressing bone resorption. Indeed, it is indisputably effective in reducing the risks associated with osteoporosis (Gossiel et al., 2016; Altman-Singles et al., 2017). The drug is effective under two working principles. On the one hand, it exerts cytotoxicity to osteoclast; on the other hand, it induces osteoblasts to secrete osteoclast inhibitory factors for coerced suppression in bone resorption. Above all, the drug itself also demonstrates high affinity to calcium phosphate and its adsorption to the surface of hydroxyapatite crystals shall prevent the dissolution of bone calcium. Overall, it is assumed that administration of Alendronate during pregnancy should prevent maternal bone resorption. Therefore, Alendronate administration in the pregnancy phase can be a means to block maternal bone resorption to monitor probable consequences in fetal bone development.

Importantly, we confirmed that calcium released by maternal mobilization is directly involved in fetal bone development. Bone histomorphometry confirmed a decrease in rat maternal bone mass after gestation, while such loss can be prevented by Alendronate administration for its action in inhibiting maternal bone resorption. However, reduced maternal bone resorption during pregnancy is accompanied by reduced fetal bone length which appears detrimental to fetal bone development.

## Materials and Methods

### *In vivo* Calcium Label and Tracing

The calcium tracker calcein [10 mg/kg body weight (BW), Sigma Aldrich, U.S. No. C087-5G] alone or both calcein and tetracycline (30 mg/kg BW, Sigma Aldrich, U.S. No. T3383) were injected to 3-month-old female rats (*n* = 8) on days 8 and 7 before mating. Four rats were injected with calcein (once a day for 2 consecutive days) and four other rats were injected with calcein and tetracycline (once a day for 2 consecutive days). After confirmed mating with vaginal swab smear test, these rats were then permitted to gestation for 21 days followed by euthanasia to isolate their fetuses. Then we randomly selected two pups from each litter for sectioning. Fetal tibias were embedded into paraffin while longitudinal and transverse sections were examined under a Zeiss 1500 fluorescence microscope. We adopted the 555 nm excitation wavelength and collected signal at 603 nm emission wavelength for tetracycline (Macri-Pellizzeri et al., 2018); therefore, it appeared as red as shown in our figures. Calcein exhibited green fluorescence under emission at 514 nm and excitation at 475 nm wavelengths.

### Alcian Blue and Alizarin Red S Staining for Fetal Skeletal

The Alcian blue staining was performed to examine structural changes in cartilage during chondrocytes differentiation as previously described. Alizarin red can be used to examine ossified bone. Briefly, the fetal masses were rinsed with PBS after formalin fixation for 3 days, followed by staining with 1% acidic Alcian blue for 2 days, and five sequential 70% ethanol washes to remove residual dyes, where cartilage region shall appear blue. The samples were then counterstained when soaked in 0.5% Alizarin red S for 2 days, where the ossified bone region shall appear red.

### Measurement of Fetal Bone Length

After the fetal skeletal staining, we used Vernier calipers to measure the bone dimensions of each part of the fetal rat. The length of long bones referred to the distance between the joints at both ends. The skull and scapula are either entirely or largely composed of intramembranous bone, and their measurements are based on relative linear proportions. In fact, we used the linear markers on the skull as the basis for its measurement. We measured the distance between nasal margin and posterior fontanelle of skull. The length of the scapula referred to the straight distance from the glenoid cavity to the medial border.

### Isolation and Inducing of Osteoclasts

The osteoclasts were isolated from femur and tibia of newborn SD rats, inoculated in overnight culture containing 10% FBS and 10 ng/ml M-CSF (Peprotech, Inc.). Suspension cells were used as osteoclast precursors. By inoculating these cells into a 24-well plate at a density of 8 × 10^5^ cells/well, osteoclasts were induced to differentiate in α-MEM containing 10% FBS, 100 ng/ml RANKL (Peprotech, Inc.), 25 ng/ml M-CSF. Polynuclear osteoclasts were observed on days 4–6.

### Tartrate-Resistant Acid Phosphatase Staining

To perform TRAP staining, sterile cover slides were plated in 24-well culture plate, bone marrow mononuclear cells were seeded on the cover slides at the density of 8 × 10^5^ cells per well. After induction by 100 ng/ml RANKL and 25 ng/ml M-CSF for 4 days, cells were treated with or without icaritin for 3 days. Cover slides were then fixed in citrate/acetone solution for 10 min and rinsed thoroughly with deionized water immediately afterward. Tartrate-resistant acid phosphatase (TRAP) staining was performed according to the manufacturer’s instruction of the TRAP staining kit (Sigma Aldrich, U.S. No. 387). Briefly, the cover slides were incubated in the TRAP staining solution for 30 min at 37°C in dark. The reaction was quenched by washing off the staining solution with deionized water, followed by mounting with cover slip after air-drying. Acid phosphatase activity appeared as purple to dark red granules in the cytoplasm of multi-nucleated, mature osteoclasts.

### Bone Resorption Assay

Long bones were segregated into small pieces using a Buehler Isomet low speed saw and trimmed to a thickness of approximately 0.3 mm per slice with diamond wafering blade. The bone slices were then soaked in 70% ethanol overnight for sterilization. These slices were rinsed with sterile water followed by air-drying in flow hood. Sterile bone slices of 2 cm^2^ each were placed in culture wells and overlaid with 8 × 10^5^ cells per well of BMMNCs in 1 ml culture medium, followed by induction with 25 ng/ml M-CSF and 100 ng/ml RANKL for 3–4 days. Alendronate treatment commenced on mature osteoclasts for 14 consecutive days. Slices were then fixed with 1% paraformaldehyde, while unattached cells were removed under 15 s bursts of sonication twice in concentrated ammonia solution. These bone slices were then stained in 1% Toluidine blue (Sigma, United States) for 10 s with three rinses in tap water. Images were captured on slides using the built-in CCD of the dissection microscope at 5× objective magnification (Carl Zeiss 006654) (Lu et al., 2017).

### Animals and Experimental Design

Female Sprague Dawley rats (*n* = 30), 250 g ± 16 g BW, 3 months old (Guangdong Province Medical Experimental Animal Center, China, SPF animals SCXK2013-0002) were given an adaptation period of 10 days with similar housing and feeding conditions prior to any treatment to minimize impact from environmental variance. Each rat was housed individually in a temperature-controlled room (24 ± 1°C, humidity 68%) with a 12-h light and dark cycle with free access to water and complete pellet diets. Rats were randomly divided into three groups, including the control group (Ctrl, non-pregnant age-matched rats, saline 1 ml/twice per day by p.o.), the pregnant group (Pregnant rats, saline 1 ml/twice per day by p.o.), and the Alendronate group (Pregnant rats, Alendronate 12 mg/kg/twice per day by p.o.). The administration period was 28 days in total (starting from 1 week before mating), until delivering their pups by surgery at term (embryonic Day 21).

### Autopsy and Sample Preparation

All 30 experimental subjects were labeled twice with calcein under a subcutaneous double injection regime of 10 mg/kg body weight per day for two consecutive days, i.e., 13–14 days and 2–3 days before animal sacrifice (Sass et al., 1997; Li et al., 2003). As mentioned previously, calcein as a fluorochrome with strong calcium affinity exhibited green fluorescence under emission at 514 nm and excitation at 475 nm wavelength is ideal in bone labeling. The use of calcein is proven valuable in the histomorphometric evaluation of dynamic parameters bone modeling and remodeling. Rats were sacrificed by heart puncture under 10% chloral hydrate (400 mg/kg body weight). The proximal tibia metaphyses (PTM) and middle part of tibia shaft (TX) were removed, anatomized to achieve free of soft tissue, thereafter embedded in methyl methacrylate and then sectioned by hard tissue Microtome (Leica 2155, Germany). The slices were prestained with Masson–Goldner Trichrome bone stain with method described as previously reported (Sun et al., 2010).

### Bone Histomorphometric Analyses

Measurements were performed with a digital system consisting of fluorescent microscope with bright-field channels. The system was coupled to a computer with a morphometry program “Bioquant OSTEO 2009” (Bioquant Corporation, United States). Static parameters, including total tissue volume (TV), trabecular bone volume (BV), and perimeter were measured and used to calculate the trabecular bone area percentage (%Tb.Ar) and trabecular thickness (Tb.Th), number (Tb.N), separation (Tb.Sp) and osteoblast number (Ob.N), osteoclast number (Oc.N), osteoblast number per mm on trabecular bone surface (%Ob.Pm), and osteoclast number per mm on trabecular bone surface (%Oc.Pm). Dynamic measurement parameters included single- and double-labeled perimeters and also inter-labeled width. The dynamic measurement parameters were used to calculate bone formation and bone resorption parameters including the percentage of labeled perimeters (%L.Pm), mineral apposition rate (MAR), trabecular bone formation rate (BFR/BV, BFR/TV, BFR/BS), trabecular absorption rate (%E.Pm), cortical bone endosteal formation rate (E-BFR/BS), cortical bone periosteal formation rate (P-BFR/BS), and cortical bone endosteal absorption rate (E-%E.Pm). Label escape correction was used for the calculation of BFR.

### Biomarkers Measurement

Blood samples were taken at the time of clinical assessment and samples of sera were kept frozen at −80°C until performing the assay. Pyridinium was measured using ELISA kit (Shanghai Bang Yi, China, No. BYE20184). N-terminal propeptide of type I Procollagen (PINP, Roche, Switzerland, No. 03141071190) and osteocalcin (OC, Roche, Switzerland, No. 12149133122) were measured using specific electro-chemiluminescence immunoassay kit in a COBASE 411 Roche analyzer.

### Statistical Analysis

All statistical analyses were performed using SPSS 5.0 software (IBM, Armonk, NY, United States) and one-way analysis of variance. The Student–Newman–Keuls (SNK) test for *post hoc* pairwise comparisons was adopted when three groups of data were compared. *P* < 0.05 was considered statistically significant.

## Results

### Maternal Calcium Deposits in Fetal Bones

**FIGURE 1:**
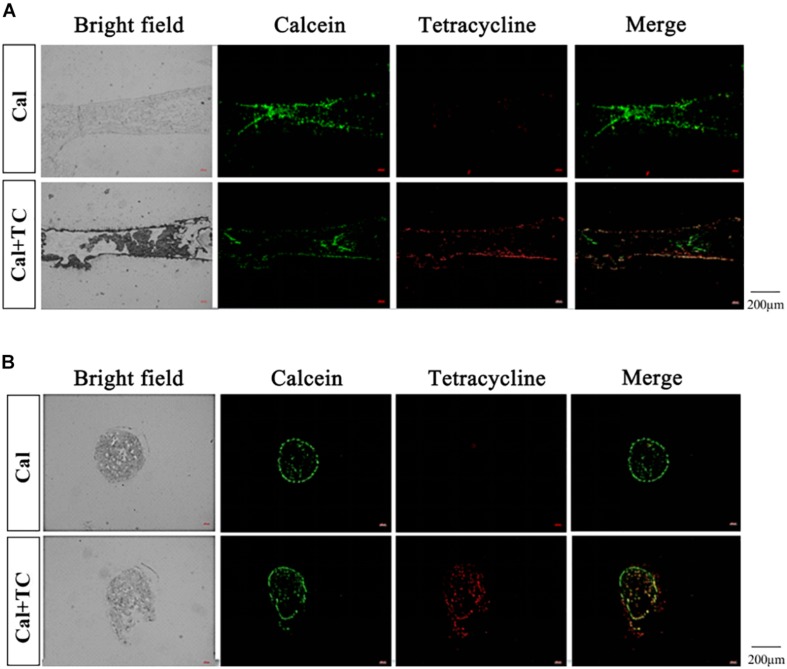
Fetal tibia fluorescence detection. **(A)** The fetal tibia longitudinal section (Cal: injected with calcein, *n* = 8; Cal + TC: injected with calcein and tetracycline, *n* = 8). **(B)** The fetal tibia transverse section (Cal: injected with calcein, *n* = 8; Cal + TC: injected with calcein and tetracycline, *n* = 8). Calcein exhibits green fluorescence (Em: 514 nm/Ex: 475 nm) and tetracycline shows red fluorescence (Em: 603 nm/Ex: 555 nm).

Both calcein and tetracycline were used to label calcium in the maternal bone, where the labeling was performed 1 week before mating, ensuring that the clearance of these two fluorescent markers from maternal circulation before pregnancy. We performed longitudinal and transverse section analysis of the fetal tibia. All the fetal bone sections of eight pups for whose mother had been injected calcein were captured with green fluorescence; for those whose mother had been injected with both calcein and tetracycline, all eight pups were labeled green despite only four among them were also labeled red. The above result reinstated that calcein can better track calcium transfer between mother and fetus than tetracycline. More importantly, these fluorescent signals mainly localized at the fetal cortical bone (Figure 1). This indicated that the fluorescent-labeled calcium deposited in the mothers’ bones is released during pregnancy and is directly recruited to assist fetal bone development.

### Alendronate Prevents Osteoclast Division and Bone Resorption

**FIGURE 2:**
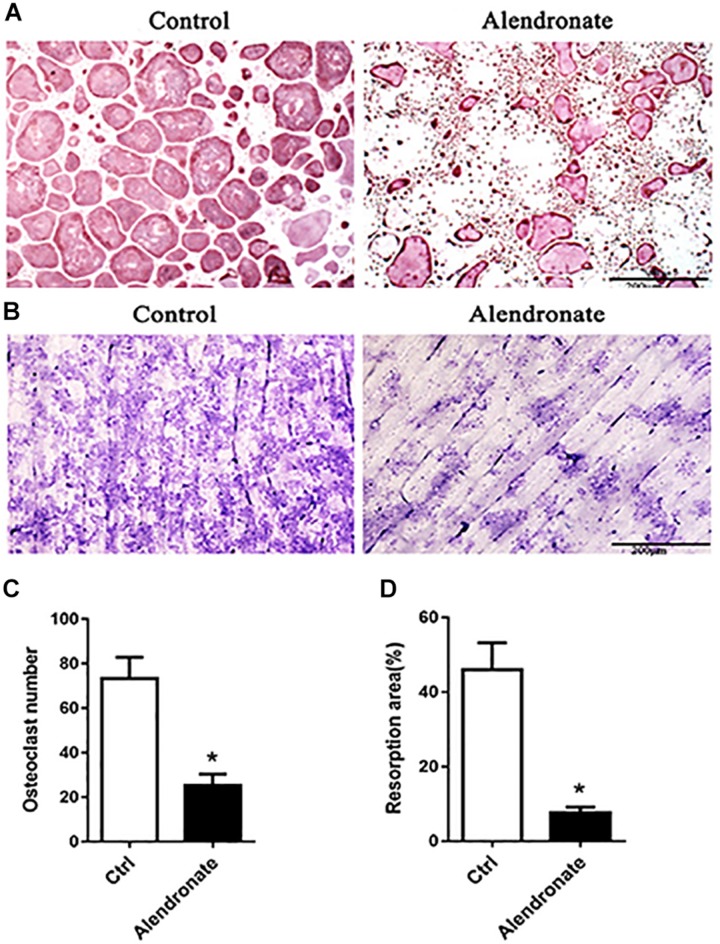
The effect of alendronate treatment to osteoclast formation and its bone resorption efficiency. **(A)** TRAP + osteoclast cells stained in purple-red (TRAP staining, 5×). **(B)** Bone resorption lacunae stained in purplish blue patches (Toluidine Blue staining, 5×). **(C)** Bar chart of the effect of alendronate treatment to osteoclast number. *P < 0.001 vs. control group. **(D)** Bar chart of alendronate treatment to osteoclast bone resorption. *P < 0.001 vs. control group.

We sought to adopt Alendronate to inhibit maternal bone resorption *in vivo.* Therefore, we first confirmed the effect of Alendronate on osteoclasts *in vitro*. As indicated in Figures 2A,C, there was a decrease in staining for osteoclasts that are positive for TRAP under the treatment with Alendronate. The formation of absorption lacuna on bone slices is a result of bone resorption of osteoclasts, while the abundance and dimensions of the lacunae provide estimations to osteoclast bone resorption function (Figure 2B). After treatment with Alendronate, the number of bone resorption lacunae was significantly reduced (Figure 2D). These results indicated that Alendronate inhibited osteoclast maturation and inhibited their bone resorption function *in vitro*.

### Effect of Alendronate on Body Weight of Rats and Fetal Rats

**FIGURE 3:**
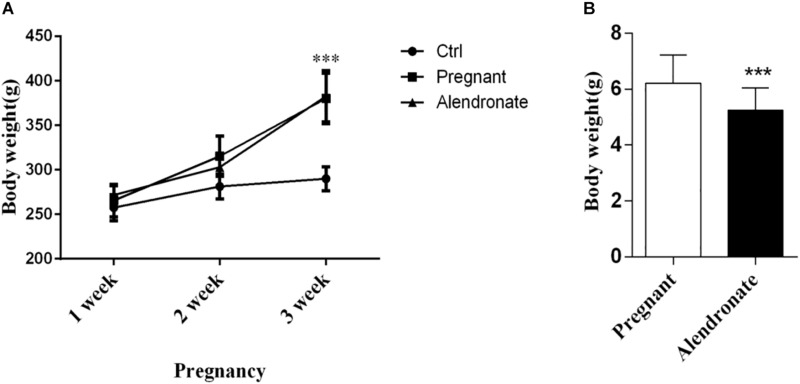
Effect of Alendronate on body weight of rats and fetal rats. **(A)** Changes in maternal and control rats body weight along pregnancy. ***P < 0.001 Pregnant group vs. Alendronate group. **(B)** Comparisons of average body weight of fetal rats in Pregnant group (*n* = 83, 10 l) and Alendronate group (*n* = 77, 10 l) at week 3. ^∗∗∗^*P* < 0.001 Pregnant group vs. Alendronate group.

We observed changes in maternal and fetal weight throughout the experiment. There was a trend of increase in weight for both pregnancy groups in the first 2 weeks compared to non-pregnant control, while there was significant increase in body weight starting from the 3rd week (*P* < 0.05, Figure 3A). When comparing between the body weight of the fetus of both the Pregnant group and Alendronate group, there was a significant reduction of fetal body weight after Alendronate treatment (*P* < 0.05, Figure 3B).

### Alendronate Decreases Cancellous Bone Loss of PTM During Pregnancy by Inhibiting Bone Resorption

**FIGURE 4:**
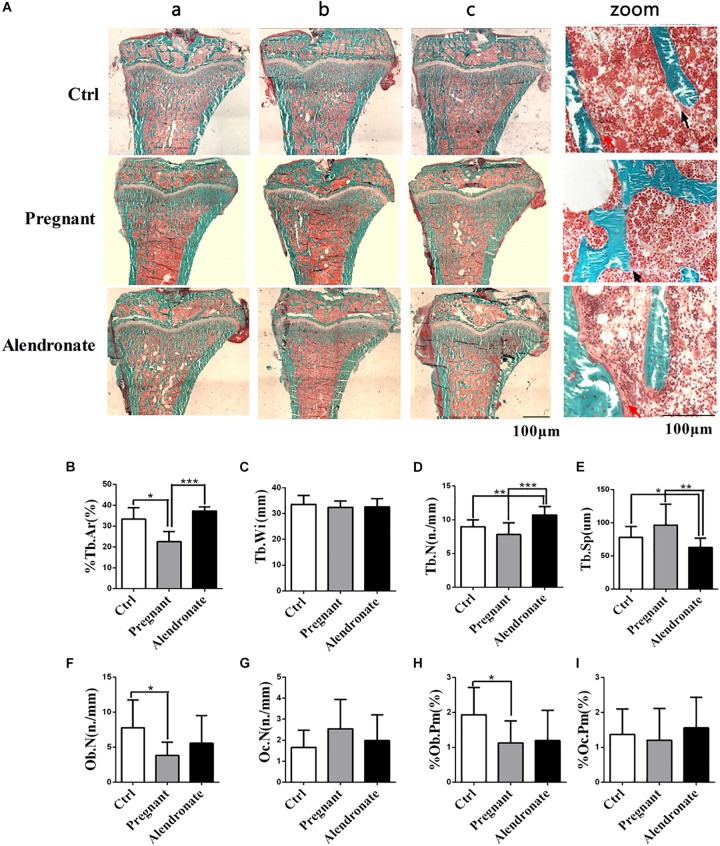
Group comparisons in static parameters of maternal rats PTM. **(A)** Longitudinal un-decalcify sections of rat tibia epiphysis with Masson–Goldner Trichrome staining. (a–c) Representative images from different animals in the same treatment group. (Zoom) Higher magnification images of rat tibia epiphysis. The area stained in red is medullary region, area in green is trabecular region and compact bone mass; red arrow indicates osteoblast, while black arrow indicates osteoclasts respectively. **(B–I)** Static parameters of PTM. *P < 0.05, **P < 0.01, ***P < 0.001 (%Tb.Ar, trabecular area; Tb.Wi, trabecular width; Tb.N, trabecular number; Tb.Sp, trabecular separation; Ob.N, osteoblast number; Oc.N, osteoclast number; %Ob.Pm, osteoblast number per mm on trabecular bone surface; %Oc.Pm, osteoclast number per mm on trabecular bone surface).

Using bone histomorphometry dynamics and static parameters, we were able to investigate the effect of Alendronate on maternal bone resorption. Bone mass was significantly reduced when animals were pregnant (Figure 4A). Animals given Alendronate demonstrated partial restoration in total bone mass compared to those without drug treatment. There was a significant decrease in %Tb.Ar when comparing Pregnant group to Ctrl group, which demonstrated a decrease in cancellous bone mass (Figure 4B). In contrast, there was significant increase in %Tb.Ar and Tb.N (*P* < 0.001), while a significant decrease in Tb.Sp (*P* < 0.01), which indicated a restoration of the static bone parameters in animals given Alendronate to levels comparable to that of Pregnant group (Figures 4C–I).

**FIGURE 5:**
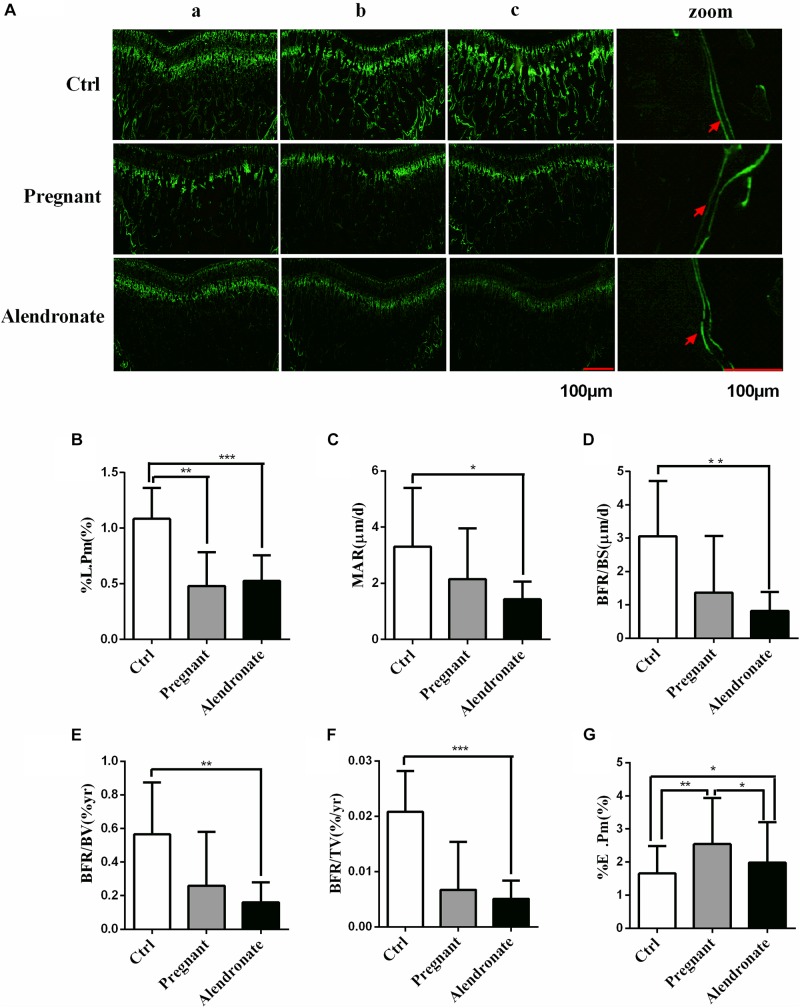
Group comparisons in dynamic parameters of maternal rats PTM Green labels compact bone and trabecular, red arrow indicates double fluorescence. **(A)** The fluorescence image of PTM, whereas (zoom) is the higher-magnified microscopic image, the red arrows indicated the positions of double calcein fluorescence label. (a–c) Representative images from different animals in the same treatment group. **(B–G)** Dynamic parameters of PTM. ^∗^*P* < 0.05, ^∗∗^*P* < 0.01, ^∗∗∗^*P* < 0.001 (%L.Pm, labeled perimeter; MAR, mineral apposition rate; BFR/BS, trabecular bone surface-based bone formation rate; BFR/BV, trabecular BV-based bone formation rate; BFR/TV, total TV-based bone formation rate; %E.Pm, trabecular absorption rate).

For the bone formation dynamic parameters of PTM (Figures 5A–F), the %L.Pm was significantly lower in the Pregnant group compared to Ctrl (*P* < 0.01). Moreover, there was substantial decreases in bone formation dynamics such as %L.Pm, MAR, BFR/BS, BFR/BV, BFR/TV, etc. when comparing Alendronate group to that of Ctrl group (*P* < 0.05). There were however no significant differences in these parameters between the Pregnant groups and Alendronate group.

For the bone resorption dynamic parameters, %E.Pm was significantly increased in the Pregnant group compared to Ctrl (*P* < 0.01). However, %E.Pm was significantly reduced in Alendronate group when compared to the Pregnant group (*P* < 0.05). These results suggest that during the late phase of gestation, the cancellous bone mass of rats would be significantly reduced, while bone formation in the pregnant rats was significantly down-regulated. Nonetheless, bone resorption at that stage would remain active. Alendronate is therefore a effective drug in preventingbone mass decline, probably by inhibiting bone resorption (Figure 5G).

### Alendronate Maintains Bone Formation in the Maternal Cortical Bone (TX) and Inhibits Its Bone Resorption

**FIGURE 6:**
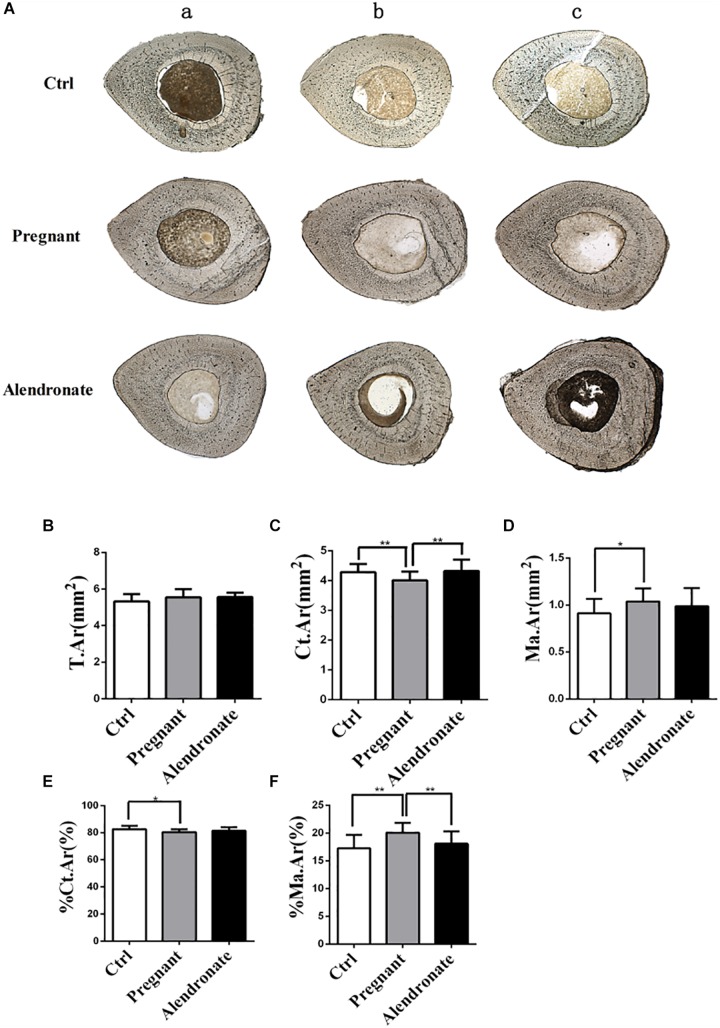
Changes in static parameters of cortical bone with or without Alendronate treatment in pregnant mice. **(A)** Horizontal cross-section of TX. The outer layer is cortical bone while the inner core is bone marrow. (a–c) Representative images from different animals in the same treatment group. **(B–F)** Static parameters of tibia diaphysis. *P < 0.05, **P < 0.01 (T.Ar, Total tissue area; Ct.Ar, cortical bone area; Ma.Ar, marrow area; %Ma.Ar, percentage of medullary area; %Ct.Ar, percentage of cortical bone area).

Pregnant group demonstrated reduced cortical bone area (Ct.Ar) and %Ct.Ar when compared to Ctrl group (Figures 6A–C,E). There was also an increase in Ma.Ar and %Ma.Ar, these indicated that there was a thinning in cortical bone mass and increase in medullary cavity (Figures 6D,F). By contrast, the Alendronate group showed maintained Ct.Ar while %Ma.Ar was reduced; there was rather no significant difference in these parameters between Alendronate group and Ctrl group, therefore Alendronate treatment indeed repressed maternal cortical bone loss caused by physiological adaptations occurring during pregnancy.

**FIGURE 7:**
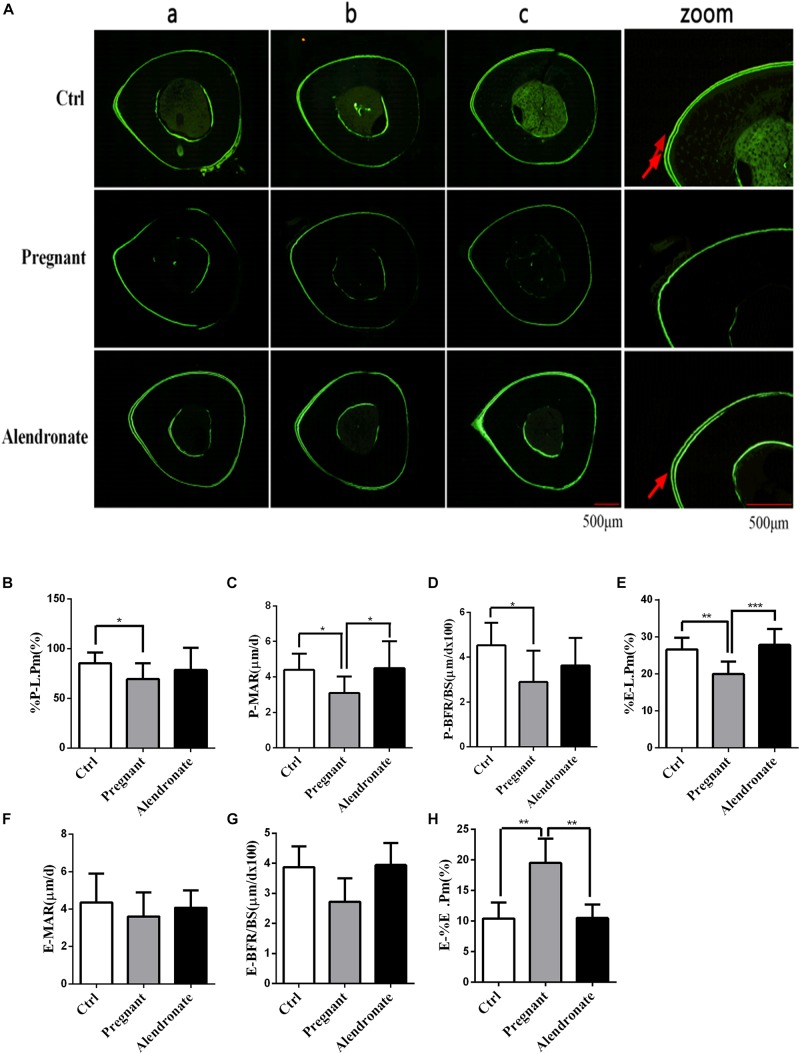
Changes in dynamic parameters of cortical bone with or without Alendronate treatment in pregnant mice. **(A)** Fluorescent image indicating horizontal cross-section of TX. Panel (zoom) is the zoom-in image of (b) while the red arrows indicated the positions of double calcein fluorescence label. (a–c) Representative images from different animals in the same treatment group. **(B–H)** Dynamic parameters of tibia diaphysis. *P < 0.05, **P < 0.01, ***P < 0.001 (%P-L.Pm, periosteal labeled perimeter; %E-L.Pm, endosteal labeled perimeter; E-BFR/BS, endosteal bone formation rate; E-MAR, endosteal MAR; P-BFR/BS, periosteal bone formation rate; P-MAR, periosteal MAR; E-%E.Pm, endosteal bone absorption).

With reference to Figure 7, there was only a trend of decrease in these dynamic parameters while there were prominent decreases in Pregnant for %P-L.Pm, P-MAR, P-BFR/BS, and %E-L.Pm when compared to Ctrl group. These dynamic parameters indicated that there was a significant reduction in the formation of cortical bone periosteum and endosteum during pregnancy. However, there was significant elevation in P-BFR/BS, %E-L.Pm, and P-IrL.wi after treatment with Alendronate. These observable changes suggested that maternal cortical bone formation was unrestricted under the action of Alendronate while this was particularly prominent in the growth of periosteum when compared to endosteum. In fact, there is no significant change in these dynamic parameters for Alendronate group when compared to Ctrl, demonstrating that formation of cortical bone mass was restored to normal under the action of Alendronate. Bone histomorphometry results of TX indicated that Alendronate exhibited a decrease in cortical bone endosteal resorption (E-%E.Pm) coupled with an increase in periosteal bone formation.

### Alendronate Affects Maternal Serum Bio-Markers of Bone Formation and Resorption

**FIGURE 8:**
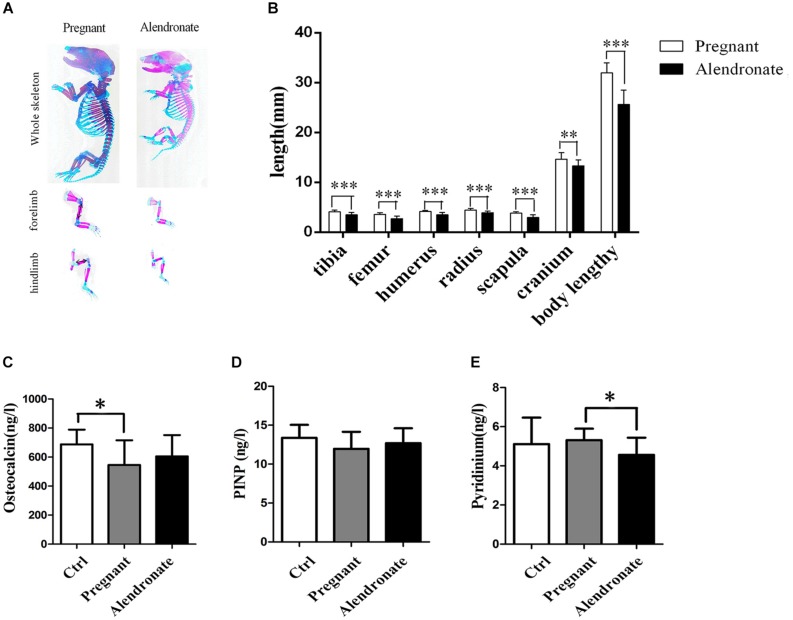
Fetal skeleton examination and maternal serum bone turnover biomarker levels. **(A)** The whole skeleton staining of fetal rats, red region stained for calcified bone structure, and blue region stained for cartilages. **(B)** The graph shows the average length of representative fetal bone structures in Pregnant group (*n* = 83, 10 l) and Alendronate group (*n* = 77, 10 l). **(C–E)** Bar-chart reporting serum levels of biomarker for bone turnover. **(C)** Osteocalcin and **(D)** PINP for bone formation and **(E)** Pyridinium for bone resorption, respectively. ^∗^*P* < 0.05, ^∗∗^*P* < 0.01, ^∗∗∗^*P* < 0.001. PINP, type I collagen amino-terminal propeptide.

We tested serum bio-markers of bone metabolism to further clarify the inhibitory effect of Alendronate on maternal bone resorption (Figures 8C–E). There was a significant reduction in OC (bone formation marker) in the Pregnant group compared to Ctrl group, while a significant reduction in pyridinium (bone resorption marker) in Alendronate group than Pregnant group. In fact, there were no significant difference in all bio-marker levels between Alendronate group and Ctrl group, which altogether suggested that there was a decrease in maternal bone formation at the late phase of gestation while there was a decrease in bone resorption under the treatment of Alendronate.

### The Fetal Bone Development Is Affected by Alendronate

As shown in Figures 8A,B, despite there was a reduction in the overall length in the fetal rat skeletal structure, the layout of the skeletal system remained unchanged under qualitative assessment, i.e., there was no abnormality in the relative spatial positioning of cartilage and calcified bone. It was estimated that the portions of long bone of the extremities stained in red remained in position but became shorter compared to the normal pregnant group, indicating a decrease in calcified bone in the long bones. After measuring the length of representative long bones, it was confirmed that there were shorter tibia, femur, humerus, radius, scapula, and cranium in the fetus after treatment with Alendronate (*P* < 0.05). Among these bone structures, the changes in the long bones of the extremities and in the spine were most obvious. These results indicated that the use of Alendronate during pregnancy had a significant impact on fetal bone development, which involved inhibiting the overall bone growth and development of the fetus, but shall not affect the relative spatial positioning between cartilage and calcified bone in fetal rat skeletal structures.

### Examining Morphological Changes to Placenta Under the Action of Alendronate

**FIGURE 9:**
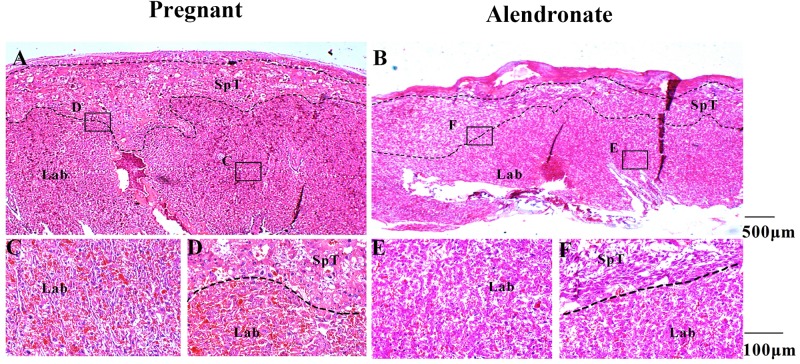
The placental structural morphology with or without Alendronate treatment. **(A,B)** H&E sections of placentas with or without Alendronate treatment. **(C–F)** Higher magnification images of panels **(A)** and **(B)**. **(C,E)** The labyrinth region while panels **(D,F)** indicated the spongiotrophoblast region.

In order to rule out the potential side-effect of Alendronate in causing placental injury which apparently also blocked calcium transmission and subsequent fetal bone development, we performed microscopic examination of the structure in the placenta. The placenta consists of three layers, including the maternal decidua, labyrinth, and spongiotrophoblast, all with distinct morphological features under the microscope. Figures 9A,B allow the comparison between Pregnant group and Alendronate group. Figures 9C–F are magnified images to the portion labeled in A and B accordingly. There is no obvious change between the groups at the maternal decidua and inner labyrinth, while there is a slightly thinner spongiotrophoblast in the group given Alendronate. Nonetheless, there is no detrimental pathological defect after the drug treatment, so there is no abnormality in placental structures during pregnancies under Alendronate treatment.

## Discussion

Studies on maternal bone metabolism and fetal bone development during pregnancy have been ongoing for more than a decade, but controversy has persisted. The major fallback is the lack of definitive proof in the source of calcium in fetal bone development during pregnancy. It is now clear that the calcium required for fetal bone development comes from maternal circulation (Kovacs, 2014; Ohata et al., 2016), yet calcium that enters the maternal circulation is largely indistinguishable from two named sources: one of which it is derived from the mother’s dietary intake of calcium; the other of which is derived from the maternal bones released by bone resorption. Previous experiments have attempted to study the relationship between fetal bone development and maternal bone metabolism by measuring the serum calcium concentration of mother and fetus or monitoring the calcium conduction of the placenta (Kaur et al., 2003; Pearson et al., 2004; O’Brien et al., 2006; Olausson et al., 2008; Jarjou et al., 2010), but these studies had not clearly explained the mechanisms between maternal bone turnover and fetal bone development during pregnancy. Whether the calcium released form maternal bones by high bone turnover shall participate in fetal bone development remains largely unknown.

There is no direct evidence for whether the calcium released from maternal bone is directly involved in fetal bone development; it was only pointed out by others that such maternal bone turnover during pregnancy is a physiological adaptation, and this physiological adaptation is essential to cope with the fetal bone development. In this experiment, we clearly confirmed that calcium released from maternal bones during pregnancy is deposited in the bones of the fetus. Such observation is suggestive of active relocation of the calcium deposited in maternal bones to play a direct role in fetal bone development.

How then, do we distinguish the calcium found in the fetal bones to whether they were sourced from the maternal bones through bone resorption, or could they have been the calcium absorbed through mothers’ diet? We adopted a fluorescent visualization approach using either calcein alone or both calcein and tetracycline together to label calcium in the maternal bone before pregnancy – both are potent calcium chelators which form stable fluorescent complexes therefore ideal as *in vivo* calcium tracers. Calcein, also known as Fluorexon, is a fluorescein-iminodiacetic acid derivative that readily label calcium deposits labeled as green (Suzuki and Mathews, 1966). Similarly, tetracycline also deposited in mineralizing tissues which shall appear red (emission 603 nm max.) under 555 nm laser excitation. We also intentionally injected both calcein and tetracycline to some other experimental subjects in order to merge two fluorescent signals together when examining the fetal tibia, which helped us to locate the deposition of those maternal calcium more accurately. We were fully aware of the issues in fluorescence compensation between signals from various fluorophores with close emission wavelengths. Nonetheless, calcein emission is clustered around green wavelengths, which may largely overlap with routine yellow tetracycline signals excited in blue-UV regions. Therefore, instead of choosing to excite tetracycline with UV wavelengths, we turned to an alternative emission spectrum of the same fluorophore with excitation near the far red region. Indeed, the signal may appear faint when excited at similar power, nonetheless substantially reduced matrix effect and sensitivity when there is less overlap in auto-fluorescent emission spectrum, and thereby adopted a lower background with higher signal-to-noise ratio when confocal images were merged. Our results showed that calcein-labeled tracking in fetal tibia was indeed better than tetracycline yet their spatial overlap is consistent with each other. On top of its common use in bone histomorphometry, their excellence in calcium tracking was fully utilized here to monitor both calcium deposition and resorption events during bone remodeling.

The rapid elimination of calcein in circulation after single-bolus injection in rabbits had been demonstrated by Golshani et al. (1993). The precaution taken by injecting calcein a week before mating could safeguard the total elimination of unbound calcein. Thus, presence of fluorescent labeling in the tibia of fetuses indicated that at least part of the calcium stemmed from resorbed maternal bone. Our current research revealed the direct participation of maternal bone calcium in fetal bone development. In this study, we have also demonstrated that chelated complexes of calcein/tetracycline and calcium shall re-enter the blood circulation with bone resorption, which then crossed the placental barrier to be integrated in fetuses. Therefore, these calcium chelators demonstrated excellent potential as tracers to study the deposition and release of calcium ions between maternal circulation and fetuses.

To further confirm that calcium released from maternal bone absorption is important for fetal bone development, we used Alendronate to inhibit maternal bone resorption during pregnancy and explored whether it will cause abnormalities in fetal bone development. We firstly confirmed changes in bone metabolism in rats during pregnancy. The process had been implicated as physiological adaptations to prepare for pregnancy in animal studies (Ohata et al., 2016). Our inflicting results seemed to conclude the ample discussions among researchers for whether there is an adaptation in skeleton turnover in the mothers. Indeed, we confirmed a decrease in both maternal cortical and cancellous bone mass during pregnancy.

The cancellous bone mass in epiphysis of the tibia is significantly lower, while the dynamic parameters also indicated a decreased bone formation and induced resorption elevated under pregnancy. In parallel, there seemed to be a coherent change in the corresponding serum biomarkers for these processes. The reduction of OC and an increase in pyridinium indicated an overall accelerated bone turnover. One study demonstrated a reductions in maternal bone during pregnancy, with the biggest documented change occurring at the lumbar spine and the trochanteric region of the hip (3–4.5% loss) (Ritchie et al., 1998). These skeletal sites are rich in trabecular bone. The results of their study are seemingly consistent with our results. It is the vast changes in trabecular bone density that revealed it is more sensitive to bone turnover.

The cortical bone (TX) had reduced bone area and increased medullary cavity which implied that rat shall first promote resorption in TX endosteum, followed by compensatory thickening in the TX periosteum. The price to pay for such compensation is a reduced bone area. Therefore, there is an actual reduction in both cortical and cancellous bone mass during pregnancy, which is in stark contrast to what was described by Zeni et al. (1999) describing no reduction in any bone mass along the gestation period. Wisser et al. (2005) otherwise used pQCT to measure women gestational changes in cortical and trabecular bone of the non-dominant distal radius. They also found a significant decrease in trabecular bone density, but cortical BV and density did not change between the first and third trimesters of pregnancy. This stark difference may be linked to differences in pregnancy burden between humans and rats, where the former is mainly singleton pregnancy, while the latter is of concomitant multiple pregnancies, so the burden of maternal rats’ bones is more substantial during pregnancy with a significant portion of bone loss.

Next, we analyzed the effects of Alendronate on maternal bone metabolism during pregnancy, and confirmed significant inhibiting effect on bone resorption. As a result, it showed a preservation of both cortical and cancellous bone mass when Alendronate was given. The cortical bone area in the mothers given Alendronate is therefore higher than those without treatment. According to the dynamic parameters of cortical bone, Alendronate can prevent bone loss during pregnancy mainly by inhibiting endosteum bone resorption and maintaining the periosteum bone formation. Our finding therefore challenged the common idea that Alendronate inhibit resorption therefore also repress bone formation (Iwata et al., 2006). This might be due to the different subjects in this study and the previous studies which mainly focus on osteoporotic animals or patients, where the subjects have distinct physiological characteristics to reach conflicting research results. However, the preservation in bone formation is limited to cortical bone, while there seemed to be a suppression in cancellous bone formation. Moreover, since there is a decrease in PYD in the Alendronate treatment group, it clearly indicated that Alendronate is effective in repressing bone resorption during pregnancy.

Fetal osteogenesis is a two-step process, there being bone formation by either an intramembranous mode or endochondral mode, followed by lengthening and thickening of compact bone. Calcium and other materials are required for osteogenesis which are supplied from the circulating blood of the mother through the placenta. Since there is a significant shortening of all fetal long bones that we examined between the groups with or without drug treatment, it clearly demonstrated that Alendronate is potent in inhibiting fetal bone development. We had also carefully checked that Alendronate did not instigate any defects in placental structures, therefore ruled out the possibility of secondary outcomes based on potential defective calcium transport across this barrier (Romano et al., 2014). It is therefore reassured that it is the reduction in maternal bone resorption which limited the supply of calcium to fetuses, which in turn restricted their skeletal growth.

Taken altogether, our results confirm that calcium released from maternal bone resorption during pregnancy is directly involved in fetal bone development. Therefore, in cases where maternal bone absorption is limited during pregnancy, fetal bone development shall be affected. Above all, this extends to an important concept that metabolic adaptation during pregnancy may actually be involved in the preparation of maternal contribution to the fetus, which is a new elaborated concept to describe the relationships between maternal bone metabolism and fetal bone development during pregnancy in mammals. Thus, our study warrants the necessity to conduct in-depth researches in this intimate and complex maternal–fetal relationship.

## Data Availability Statement

All datasets generated for this study are included in the article/supplementary material.

## Ethics Statement

The animal study was reviewed and approved by the Committee of Experimental Animal Ethics, Guangdong Pharmaceutical University.

## Author Contributions

LL and QL: study design and final version of manuscript approval. HJ, LL, LR, YL, JC, and WC: data acquisition. HJ, LL, KM, ST, JC, and GY: data analysis and interpretation. LL: drafting manuscript and responsible for the integrity of the data analysis.

## Conflict of Interest

The authors declare that the research was conducted in the absence of any commercial or financial relationships that could be construed as a potential conflict of interest.
